# Global landscape of neuromyelitis optica spectrum disorder clinical trials: trends in therapies, geography, and outcomes

**DOI:** 10.3389/fimmu.2026.1695727

**Published:** 2026-04-15

**Authors:** Gang Wang, Jun Li, Xinling Su, Zhuangwei Fang, Yuting Xu, Juan Zheng, Ning Wang, Liping Huang

**Affiliations:** Department of Rehabilitation Medicine, The First Medical Center, Chinese People’s Liberation Army (PLA) General Hospital, Beijing, China

**Keywords:** biologics, clinical trials, complement, endpoints, IL-6, neuromyelitis optica spectrum disorder

## Abstract

**Background:**

Neuromyelitis optica spectrum disorder (NMOSD) is a rare autoimmune disease for which several targeted therapies have emerged in recent years. A systematic overview of registered clinical trials can clarify development trends and research priorities.

**Methods:**

We searched the Trialtrove database for interventional NMOSD trials registered up to August 15, 2025. Eligible studies were analyzed for trial phase, status, geography, therapeutic agents, and primary endpoints.

**Results:**

A total of 141 trials met inclusion criteria. Phase I studies were most common (35.8%), while phase III trials (14.2%) exceeded standalone phase II designs (13.3%). Nearly half were completed, though 31.9% lacked public results. China (64.5%) and the United States (24.8%) led global activity. Therapeutic programs focused on B-cell depletion (34.0%), complement C5 inhibition (17.6%), and IL-6 receptor blockade (10.9%), with BTK inhibitors and other novel approaches emerging. Primary endpoints emphasized safety and relapse prevention, while visual outcomes, patient-reported measures, and biomarkers were rarely included.

**Conclusion:**

Taken together, these findings illustrate the rapid expansion of NMOSD clinical research, alongside persistent gaps in transparency and trial design. NMOSD research has expanded rapidly, driven by biologics and regulatory momentum. Future trials should strengthen transparency, address recruitment challenges, and broaden outcome measures to better reflect patient needs.

## 1 Introduction

Neuromyelitis optica spectrum disorder (NMOSD) is a rare autoimmune disease characterized by multifocal central nervous system (CNS) inflammation, predominantly affecting the optic nerves and spinal cord, and typically manifesting as attacks of visual loss and paralysis. Approximately 90% of patients experience a relapsing course, recovery from relapses is variable, and repeated inflammatory episodes can result in permanent disability or even death (1). Within five years of disease onset, 41% of patients become legally blind and 22% require mobility aids (2). Reported prevalence and incidence rates range from 0.07 to 10 and 0.029 to 0.880 per 100,000 population, respectively (3). Most of the cases are positive for autoantibodies targeting the water channel aquaporin-4 (AQP4-IgG). Activated B and T cells, innate immunity cells, pro-inflammatory cytokines, and activated complement contribute to the formation of the NMOSD lesions (4). According to the affected regions of the CNS, there are six core clinical characteristics in NMOSD: optic neuritis (ON), acute myelitis, area postrema syndrome, acute brainstem syndrome, symptomatic narcolepsy or acute diencephalic clinical syndrome, or symptomatic cerebral syndrome (5). The exact etiology of NMOSD remains unclear. Although factors such as diet, low vitamin D levels, infections, and vaccine exposure have been investigated, no causal association has been established, and further research is warranted (6). Currently, there is no known curative treatment for NMOSD; therefore, the main goals of therapy are to counteract acute attacks promptly and effectively and to prevent future attacks by initiating immunotherapy as soon as a definite diagnosis of NMOSD is established (7). Previously, the primary treatments available for NMOSD included corticosteroids, plasma exchange, immunoadsorption, and immunosuppressants (such as azathioprine, mitoxantrone, mycophenolate mofetil, and others). High-dose intravenous glucocorticoids are utilized for acute relapse management; long-term relapse prevention relies on maintenance immunosuppressants or targeted biologics (8). Due to the absence of targeted therapies, NMOSD management has long posed a challenge for clinicians. A 2022 publication noted that “early treatment for acute attacks is highly recommended, although there are no randomized controlled trials on acute treatments.” Prior to June 2019, no NMOSD-specific therapies had been approved by regulatory authorities worldwide (9). Beginning in 2019, the FDA approved eculizumab (2019), inebilizumab (2020), and satralizumab (2020) for NMOSD. This regulatory milestone spurred a sharp increase in related studies, such that 2019 has been described as the “year of NMOSD” (10). Nevertheless, a PubMed search using the terms “neuromyelitis optica spectrum disorders,” “randomized,” “controlled,” and “acute” identified only one randomized controlled trial addressing acute NMOSD, published in October 2022. This highlights that therapeutic research in NMOSD remains in its early and exploratory stages. Despite NMOSD being a rare dis ease, its socioeconomic impact is enormous, particularly on health care systems in developing countries.

Given the heterogeneity of its clinical course, and the recent but limited availability of targeted therapies, there is an urgent need to systematically evaluate the landscape of clinical trials in this field. To date, most available data derive from small-scale or multicenter efforts, and gaps remain regarding trial design, drug development priorities, and outcome measures. By analyzing registered interventional studies from the largest global clinical trial repository, this work provides a comprehensive overview of therapeutic strategies under investigation and demonstrates emerging trends. Such insights are critical for clinicians and researchers to anticipate future treatment directions, optimize trial design, and ultimately translate novel therapies into improved patient care.

## 2 Methods

### 2.1 Data source and selection criteria

This study used data from the Trialtrove database, a global repository that aggregates trial information from multiple registries worldwide. We searched for all trials registered up to August 15, 2025, related to the treatment of NMOSD. The search terms were “Disease: ‘neuromyelitis optica spectrum disorders’ OR MeSH Term: ‘neuromyelitis optica’.” To ensure data relevance and reliability, only interventional studies were included.

### 2.2 Inclusion and exclusion criteria

To ensure comprehensive coverage, our preliminary screening included all trials related to the treatment of NMOSD. The inclusion criteria comprised interventional trials explicitly focused on pharmacotherapy, with clearly defined therapeutic targets relevant to NMOSD. Exclusion criteria applied to studies that lacked critical information (e.g., therapeutic approach), contained incomplete datasets, or were non-interventional in design (such as observational studies or registries that did not directly assess therapeutic outcomes). Trials without sufficient detail to determine the therapeutic target were also excluded from the final analysis.

### 2.3 Data extraction, quality control, and statistical analysis

Because the database contained a large number of information fields, we selected seven key variables for analysis: start date, trial phase/status, geographic distribution, primary tested drug, drug mechanism of action, and primary endpoint. Two investigators independently extracted trial-level characteristics using a predefined protocol, including trial ID, start year, country/region, phase, status, geographic distribution, primary tested drug, drug mechanism of action, and primary endpoint. All extracted fields were cross-verified against multiple databases to ensure accuracy and completeness. Any discrepancies were resolved through consensus-based discussion. Key characteristics were summarized and presented in figures.

## 3 Results

By August 15, 2025, a total of 141 trials met the criteria for statistical analysis. The relevant information was systematically analyzed and summarized.

### 3.1 Distribution and temporal trends of clinical trial phases

**Figure 1:**
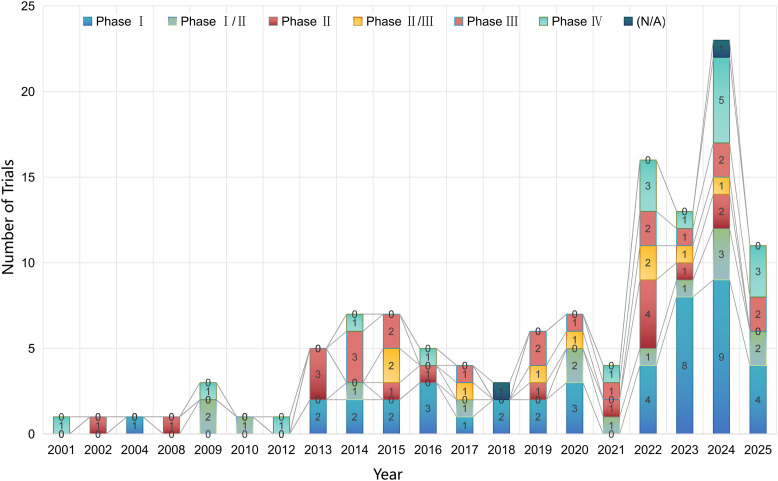
Trends in newly initiated clinical trials by year and trial phases.

Because several trials in the planning phase had not yet determined their start dates, this section reports statistics only for the 120 trials with complete information on both start dates and trial phases. Phase I trials predominated, constituting the largest proportion (35.8%) of all registered studies. Notably, phase III trials (14.2%) were more common than phase II standalone trials (13.3%). Combined phase trials (I/II: 12.5%, II/III: 7.5%) comprised a significant minority. Phase IV trials represented 15.0% of the total, while a small number (1.7%) had unspecified phases (N/A). A marked upsurge in overall clinical trial initiation occurred from 2018 onwards, with the years 2024 (projected: 23 trials, 19.2%) and 2025 (projected: 11 trials, 9.2%) exhibiting the highest yearly totals within the dataset. Prior to this period (2010-2017), yearly initiation remained comparatively low. These data demonstrates a recent, substantial increase in research activity focused on NMOSD treatments, primarily driven by phase I investigations (**Figure 1**).

Among the 17 phase III NMOSD trials, 9 (52.9%) were randomized controlled trials (RCTs) and 8 (47.1%) used non-randomized controlled designs. Within the RCTs, 5/9 (55.6%) employed placebo controls, whereas 4/9 (44.4%) used non-placebo comparators (e.g., active control or other non-placebo control strategies). Among non-randomized phase III trials, 6/8 (75.0%) were single-arm studies, while 2/8 (25.0%) incorporated non–single-arm non-randomized comparators. Overall, these data indicate that phase III NMOSD evidence generation is split between classical randomized approaches and feasibility-oriented designs (including single-arm studies), underscoring the importance of interpreting “Phase III” labels in conjunction with control strategy and allocation method.

### 3.2 Trial status and barriers to completion

**Figure 2:**
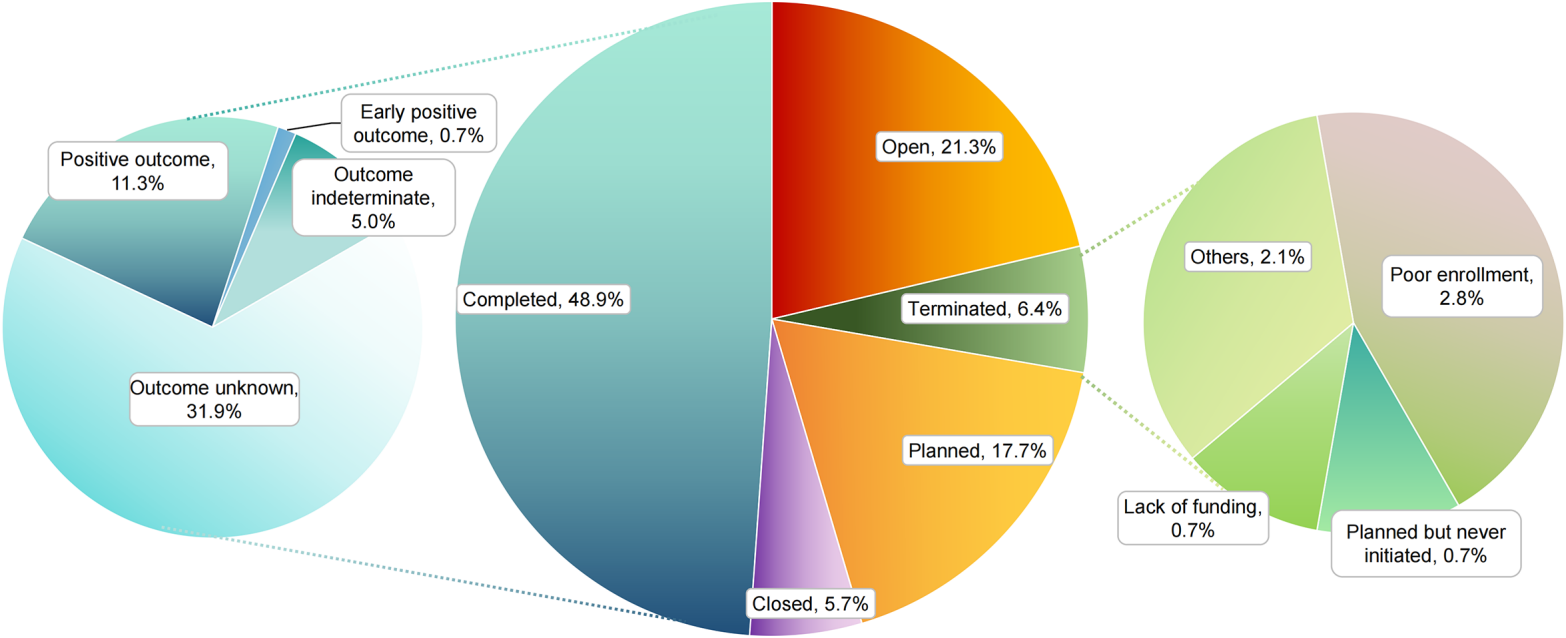
Distribution of trial status for NMOSD therapeutics.

Among all registered trials, 48.9% had reached completion, underscoring a considerable level of research activity. Nevertheless, outcome transparency remains limited: 31.9% of completed trials did not provide publicly accessible results (status: Completed, Outcome Unknown), and an additional 5.0% reported results that were classified as indeterminate. Ongoing recruitment (Open status) accounted for 21.3% of trials, while 17.7% were still at the planning stage. Discontinuation was documented for 17 trials (12.1% of the total). Where reasons were specified, inadequate patient enrollment (Poor enrollment) was most frequently cited (2.8% of terminated trials with reported reasons), followed by unspecified Other causes (2.1%) and insufficient funding (0.7%). A further 5.7% of studies were categorized as Closed without additional details. Poor enrollment and insufficient funding likely reflect structural constraints of rare-disease trials. In NMOSD, limited patient pools, restrictive eligibility criteria, and competition with parallel studies can slow accrual, while prolonged recruitment increases operational costs (monitoring, drug supply, and long follow-up), raising the risk of funding-related discontinuation. Collectively, these findings highlight both the progress achieved and the persistent challenges in trial execution and result dissemination (**Figure 2**).

### ​3.3 Geographical trial concentration​

**Figure 3:**
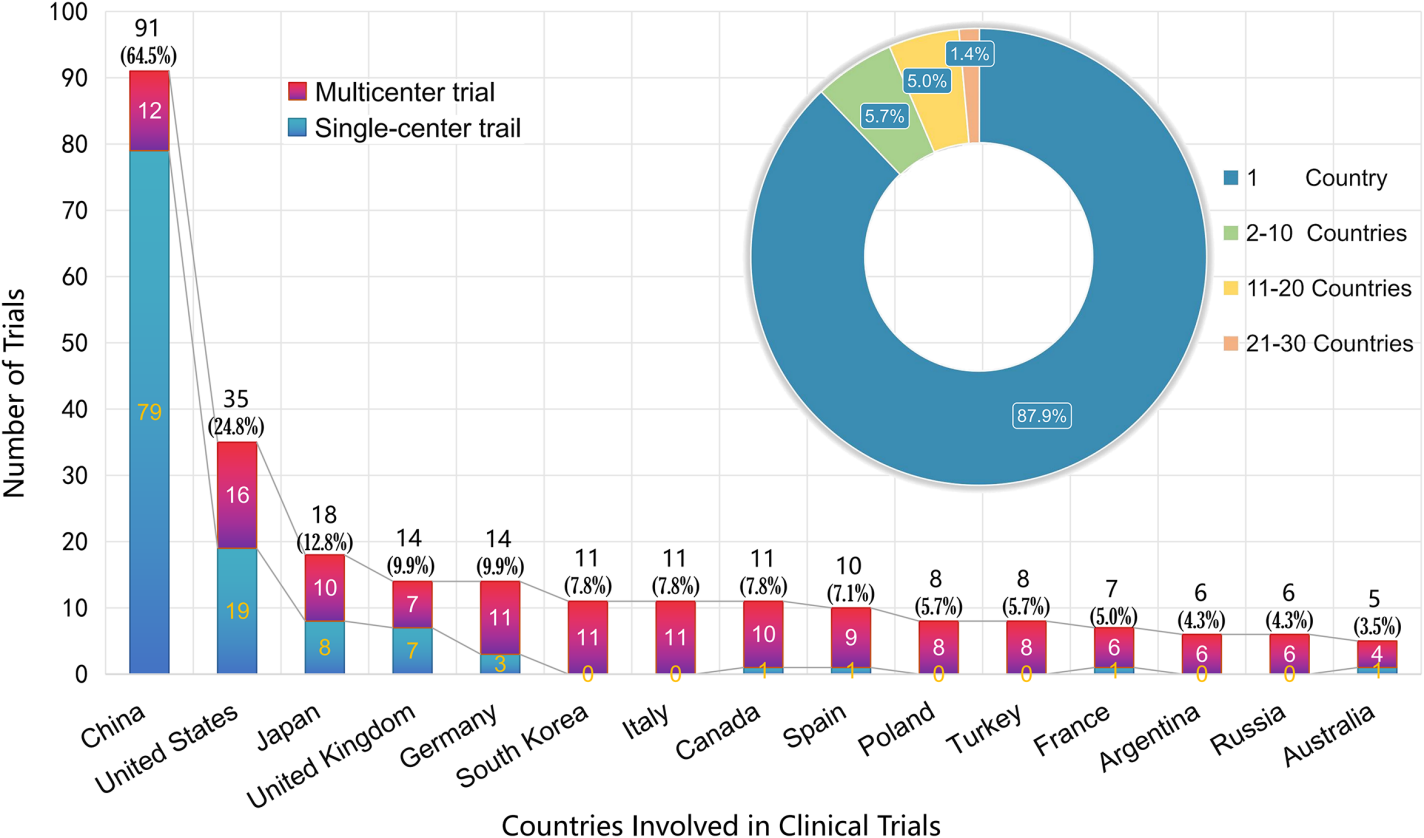
Geographic distribution of NMOSD clinical trials.

Across the registered clinical trials for NMOSD therapies, China contributed the largest share, accounting for 91 trials in total (64.5% of all studies), most of which were led independently (79 single-country trials) with additional participation in 12 multicenter efforts. The United States followed with 35 trials (24.8%), including 19 led domestically and 16 in collaboration with other nations. Japan contributed 18 trials (12.8%), nearly evenly split between independent and multicenter studies, while Germany and the United Kingdom each participated in 14 trials (9.9%), again with a balance between domestic leadership and multicenter collaboration. A group of other countries showed meaningful but smaller involvement, including South Korea, Italy, and Canada (11 trials each, 7.8%), Spain (10 trials, 7.1%), and Poland and Turkey (8 trials each, 5.7%). France, Argentina, Russia, and Australia contributed fewer trials, ranging from 5 to 7 each. The overwhelming majority were confined to single-country studies, with 124 such trials, while only a limited number extended across borders, including 8 involving 2–10 countries, 7 involving 11–20 countries, and just 2 spanning 21–30 countries. Overall, this distribution emphasizes that research activity has been concentrated in a few leading countries, particularly China and the United States, with relatively limited multinational collaboration across the broader trial landscape (**Figure 3**).

### 3.4 Therapeutic agent prioritization

B-cell–targeted monoclonal antibodies constituted the largest therapeutic class, accounting for 34.0% of all interventions. Within this group, Inebilizumab, a CD19 monoclonal antibody, was the most frequently used agent (19.8%), followed by the CD20 antibodies Rituximab (10.9%) and Ofatumumab (3.3%). Complement pathway inhibition through C5 protein blockers represented the second most common category (17.6%), equally divided between Eculizumab (8.8%) and Ravulizumab (8.8%).

**Figure 4:**
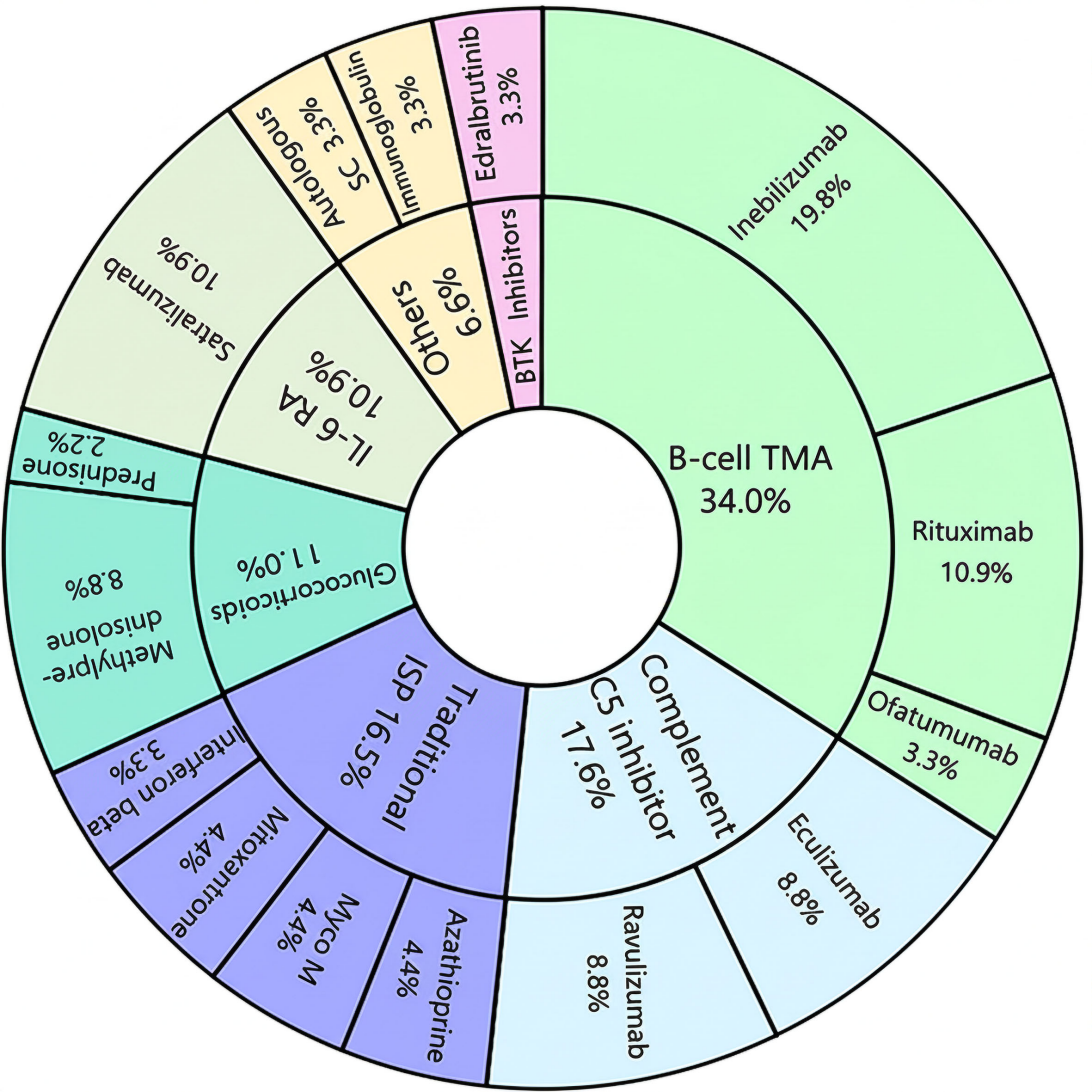
Therapeutic agents in NMOSD clinical trials by drug class.

Conventional immunosuppressants also remained relevant (16.5%), with azathioprine, mycophenolate mofetil, and mitoxantrone each contributing 4.4%, and interferon beta accounting for 3.3%. Glucocorticoids (11.0%), particularly methylprednisolone (8.8%), were frequently used for acute management. Targeted biologics included Satralizumab, an IL-6 receptor antagonist, which represented 10.9% of interventions. Bruton tyrosine kinase (BTK) inhibition was explored through Edralbrutinib (3.3%). Additional strategies (6.6%), such as autologous stem cell therapy and immunoglobulin, were also investigated, adding further diversity to the therapeutic landscape (**Figure 4**).

B-cell TMA: B-cell targeted monoclonal antibodies; ISP: immunosuppressants; IL-6 RA: IL-6 receptor antagonist; BTK: Bruton’s tyrosine kinase; Myco M: mycophenolate mofetil;Autologous CS: autologous stem cell.

### 3.5 Therapeutic target landscape in NMOSD drug development

Development programs prioritized B-cell surface markers. MS4A1 (CD20) was the most frequently investigated target (23 trials), followed by CD19 (13 trials). Complement C5 inhibition constituted another major axis (16 trials). IL-6 receptor blockade was also common (12 trials). Bruton’s tyrosine kinase (BTK) represented a key intracellular signaling target (10 trials). In addition, unspecified targets were reported in 11 trials.

**Figure 5:**
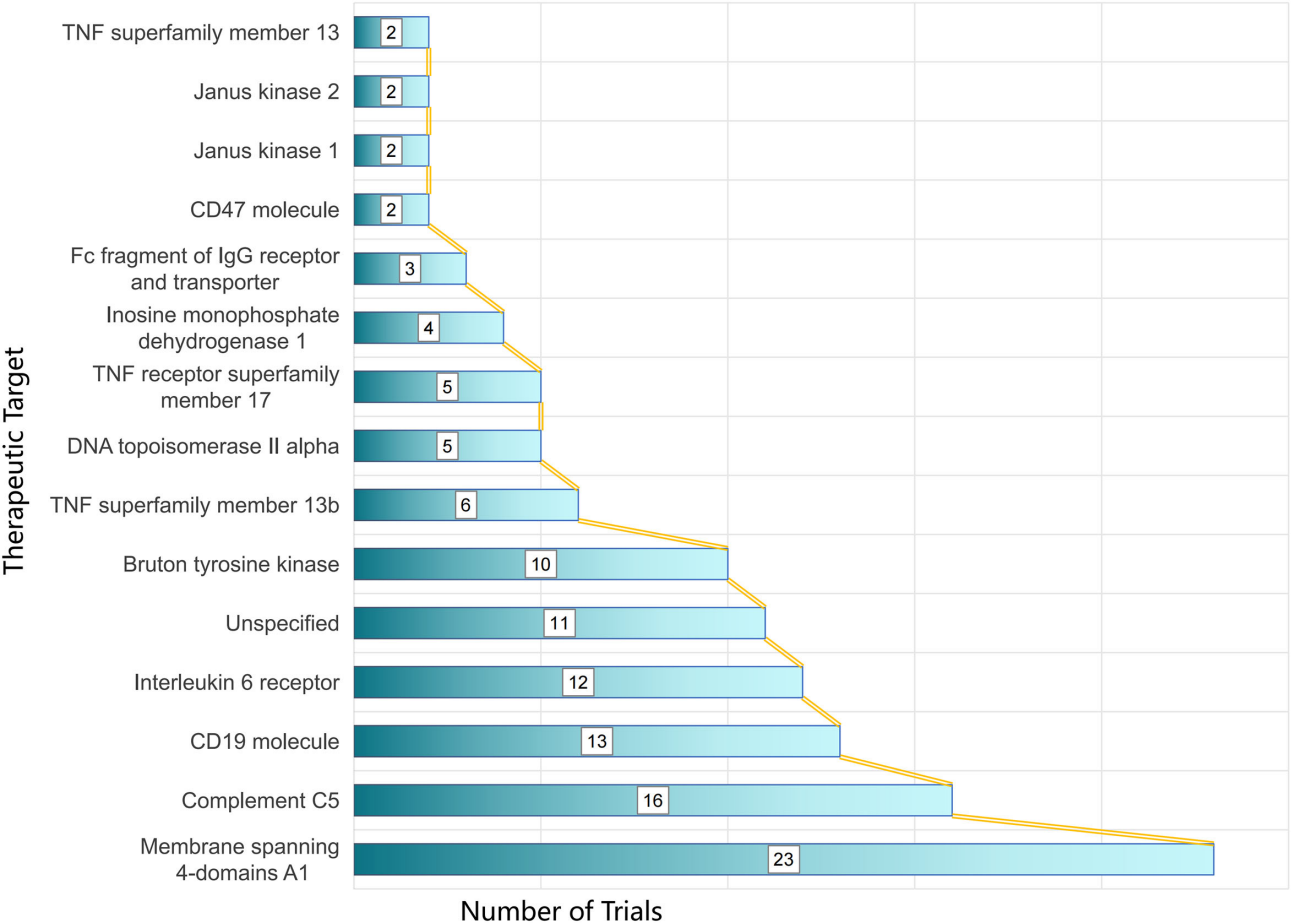
Therapeutic target landscape in NMOSD drug development.

Additional pathways included members of the TNF superfamily—TNFRSF13B (TACI) (6 trials), TNFRSF17 (BCMA) (5 trials), and TNFSF13 (APRIL) (2 trials)—as well as DNA topoisomerase IIα (TOP2A) (5 trials), IMPDH1 (4 trials), FcRn (3 trials), CD47 (2 trials), and JAK1 and JAK2 (2 trials each) (**Figure 5**).

### 3.6 Primary endpoint landscape in NMOSD drug trials

**Figure 6:**
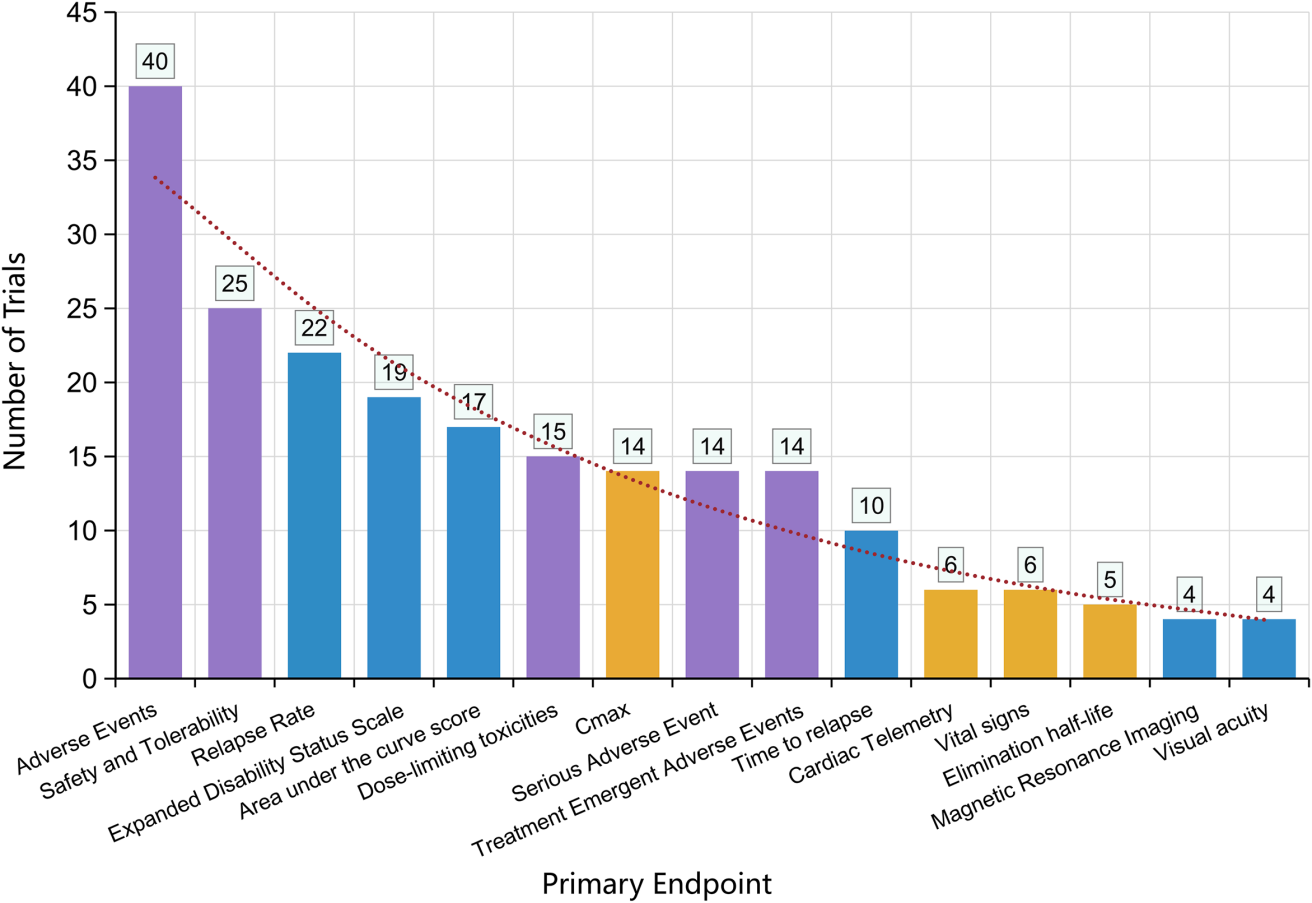
Primary endpoint landscape in NMOSD drug trials.

Safety monitoring was the most frequently selected primary endpoint. Adverse events constituted the leading measure (40 trials). Other safety-related endpoints included safety and tolerability (25 trials), dose-limiting toxicities (15 trials), serious adverse events (14 trials), and treatment-emergent adverse events (14 trials). Efficacy was primarily assessed through relapse activity, with relapse rate (22 trials) and time to relapse (10 trials) serving as key measures. Neurological disability progression, evaluated using the Expanded Disability Status Scale (EDSS) (19 trials), was another important functional endpoint. Pharmacokinetic parameters were also frequently examined, including area under the concentration–time curve (AUC) (17 trials), Cmax (14 trials), and elimination half-life (5 trials).Additional assessments included cardiac telemetry (6 trials), vital signs (6 trials), magnetic resonance imaging (MRI) (4 trials), and visual acuity (4 trials) (**Figure 6**).

### 3.7 AQP4-IgG–positive enrollment requirement

Across the 141 included interventional NMOSD trials, 54 studies (38.3%) explicitly required AQP4-IgG seropositivity for enrollment, whereas the remaining 87 trials (61.7%) either enrolled mixed serostatus populations or did not specify an AQP4-IgG-positive requirement in publicly available registry fields. This pattern indicates that a substantial proportion of NMOSD drug development is conducted in an AQP4-enriched population, while a larger segment of trials continues to evaluate therapies in broader or incompletely characterized NMOSD cohorts.

## 4 Discussion

The increase in NMOSD trial initiations after 2018—peaking in 2024 (19.2%) and 2025 (9.2%)—was temporally aligned with the approval of novel therapies beginning with eculizumab (anti-C5) in 2019 (11). This milestone established complement inhibition as a clinically effective therapeutic strategy and stimulated subsequent development efforts. The predominance of phase I trials (35.8%) was consistent with intensified exploration of new mechanisms beyond established B-cell depletion and complement pathways, including Bruton’s tyrosine kinase (BTK) inhibitors (e.g., evobrutinib), FcRn antagonists (e.g., rozanolixizumab), and stem cell therapies (e.g., hUC-MSCs), many of which entered phase I between 2023 and 2025.

The relatively higher frequency of phase III trials (14.2%) compared with standalone phase II studies (13.3%) suggests a tendency toward accelerated progression (“leapfrogging”), whereby promising agents such as ravulizumab (long-acting C5 inhibitor) advanced directly from phase II/III to phase III designs, as illustrated by the CHAMPION-NMOSD trial’s use of external placebo controls to facilitate regulatory submission. This pattern was further influenced by China’s dominant contribution to trial volume (64.5% of global studies), where regulatory reforms since 2017 have prioritized orphan drug development (12), enabling rapid advancement of candidates such as MIL62 (CD20 mAb) through combined phase Ib/III designs.

Approximately 15.0% of trials were conducted as phase IV or post-marketing surveillance studies, particularly for inebilizumab and satralizumab, indicating an increased focus on real-world data collection to evaluate long-term safety and detect rare but serious adverse events. This emphasis was especially relevant for B-cell-depleting therapies, where concerns such as progressive multifocal leukoencephalopathy (PML) cannot be fully addressed in pivotal phase II/III studies (13). Post-authorization safety initiatives-including nationwide all-case surveillance of satralizumab in Japan (14) and mandatory registries for inebilizumab in Europe and the United States-demonstrate the role of extended monitoring in documenting long-term safety profiles in broader patient populations.

By contrast, the relatively low representation of conventional phase II trials (13.3%) highlighted limitations in generating robust proof-of-concept data. This pattern suggests that adaptive trial designs may offer value by balancing the need for accelerated development with the requirement for rigorous efficacy validation.

The analysis of NMOSD clinical trial status indicates considerable research productivity while also highlighting persistent systemic challenges. Nearly half of all registered trials (48.9%) reached completion, a rate broadly comparable to other complex neurological conditions. However, 31.9% of these completed trials have not reported outcomes (“Completed, Outcome unknown”), underscoring a substantial transparency gap and echoing broader concerns regarding reporting bias, which complicates evidence synthesis (15). Here, transparency primarily refers to incomplete public availability of results in registry fields and/or the public literature, rather than implying intent. In our dataset, this is reflected by the high proportion of completed trials labeled Completed, Outcome unknown, and by trials marked Closed without clear reasons, which limits interpretability and may contribute to dissemination bias (e.g., delayed or absent reporting of neutral/negative findings).This lack of disclosure stands in contrast to international initiatives such as the WHO International Clinical Trials Registry Platform (ICTRP) and the AllTrials campaign, both of which emphasize the ethical obligation to make trial results publicly available. Addressing this transparency gap is critical to ensure that NMOSD trial data can be synthesized and translated into meaningful patient benefit. This lack of disclosure contrasts with the smaller proportion of studies (5.0%) that explicitly reported inconclusive results, suggesting systemic issues that extend beyond trial feasibility alone.

At the same time, ongoing recruitment (“Open”: 21.3%) and planning-stage activity (17.7%) reflect sustained momentum in NMOSD research. Nevertheless, the relatively high discontinuation rate (12.1%, n = 17) remains concerning. Among the subset with reported reasons, insufficient patient enrollment was the most frequent cause (2.8% of terminated trials), exceeding funding limitations (0.7%). This pattern is consistent with recruitment barriers inherent to rare diseases, where diagnostic heterogeneity and restrictive eligibility criteria pose additional challenges to patient accrual. The presence of “Closed” trials without further specification (5.7%) adds further uncertainty regarding their contribution to the evidence base. NMOSD trials are vulnerable to slow recruitment because many patients are already treated with off-label immunosuppression or approved biologics, reducing willingness to enroll in placebo-controlled or washout-required protocols. When recruitment slows, costs rise and funding pressure increases, especially for investigator-initiated studies. Pragmatic eligibility criteria, broader site networks, and adaptive designs may reduce failure risk.

Taken together, these findings point to the need for strategies that strengthen both feasibility and transparency in NMOSD trials. Priorities include (1): adoption of pragmatic and adaptive trial designs to mitigate feasibility constraints (2); development of innovative and patient-centered recruitment approaches to address enrollment bottlenecks; and (3) stronger enforcement of mandatory trial registration and results disclosure frameworks, as advocated by Pratt et al. (16). These measures are essential to maximize the translational impact of ongoing and future NMOSD research efforts.

### 4.1 Epidemiological drivers

The marked geographical concentration of NMOSD trial activity in China (64.5% of all studies) and the United States (24.8%) underscores the interplay between epidemiology, economic context, and regulatory priorities in shaping global research landscapes. Recent syntheses estimate global prevalence at approximately 0.7–10 per 100,000, with East Asian populations consistently at the higher end of this spectrum under the 2015 IPND criteria compared to 2006 definitions (3). Japan, for instance, reports a national prevalence of 5.4 per 100,000 in its 2023 clinical practice guideline, reflecting both a substantial patient pool and mature neurology subspecialty networks (17). Comparable data from South Korea document incidence and prevalence patterns with strong female predominance, supporting the feasibility of site accrual and explaining its steady, though smaller, trial contribution (18). Chinese cohorts have been shown to exhibit both high prevalence and well-developed registry systems, which, together with ethnic risk gradients observed across multi-ethnic Asian settings such as Malaysia (19), help explain why China not only leads in absolute trial numbers but also maintains capacity to sustain predominantly single-country studies (79 of 91 trials). By contrast, countries with smaller trial counts (e.g., France, Argentina, Australia) often rely on multinational consortia, given their more limited patient pools and recruitment challenges. Beyond epidemiology and infrastructure, leading countries may benefit from policy environments that facilitate trial start-up, multicenter networks, and post-approval evidence generation. Trial participation (including open-label extensions) can also function as a practical access pathway to innovative therapies when reimbursement or market availability is evolving, although continuity of access varies by sponsor policy and local health systems. When trials close early, participants typically return to standard-of-care maintenance therapy; however, access to the investigational product may be interrupted unless an open-label extension or other sponsor-supported access mechanism exists. This reinforces the importance of clear transition plans and complete safety reporting even when efficacy endpoints are not fully met.

### 4.2 Economic and infrastructure influences

Economic capacity remains a decisive determinant. High-income countries such as the United States, Japan, Germany, the United Kingdom, and Canada continue to contribute substantially, leveraging advanced diagnostic infrastructure, specialized clinical centers, and sustainable funding streams. China, though still classified as an upper-middle-income economy, has rapidly expanded clinical trial infrastructure, national registries, and rare disease policy initiatives, enabling not only a dominant share of independent studies but also increasing participation in multicenter collaborations. This pattern suggests that middle-income systems with strong policy momentum can, under the right conditions, rival or exceed high-income peers in rare disease research output.

### 4.3 Regulatory frameworks as accelerators

Finally, regulatory frameworks appear to act as accelerants of trial activity. The U.S. Food and Drug Administration set early precedents with sequential approvals of eculizumab (2019), inebilizumab (2020), satralizumab (2020), and ravulizumab (2024), while Japan’s PMDA authorized satralizumab in 2020 and the EMA followed with satralizumab and inebilizumab approvals shortly thereafter. China’s NMPA approval of satralizumab in 2021 coincided with a sharp uptick in trial registration and increasing state prioritization of orphan indications. In this context, the finding that 124 of 141 total studies remain confined to single-country efforts demonstrates both the strength of domestic trial ecosystems in leading countries and the relative underdevelopment of multinational frameworks in NMOSD research. Taken together, these patterns suggest that country-level trial participation is primarily driven by (1) disease burden and availability of identifiable cohorts, (2) macroeconomic capacity to support resource-intensive rare disease trials, and (3) regulatory and policy incentives that accelerate trial initiation and drug access. These interacting determinants explain the overwhelming dominance of China and the United States while clarifying why many other regions participate predominantly through multinational consortia rather than domestically led efforts.

B-cell–targeted monoclonal antibodies represented the largest therapeutic category (34.0%) in this analysis. Among them, inebilizumab (19.8%) was the most frequently used. As a humanized IgG1 monoclonal antibody targeting CD19, inebilizumab binds to a broader range of B-cell lineages-including pre-B cells and a subset of plasma cells-compared with CD20-directed agents. This broader expression profile allows for more effective depletion of circulating plasmablasts, a key source of pathogenic AQP4-IgG, and underpins its therapeutic role in B-cell-mediated autoimmunity (20). Its clinical efficacy was demonstrated in the phase II/III N-MOmentum trial (NCT02200770), in which only 12% of patients receiving inebilizumab experienced a relapse, compared with 39% in the placebo group, confirming its ability to reduce NMOSD relapse risk (13). Other B-cell–directed therapies, such as rituximab (10.9%) and ofatumumab (3.3%), also contributed substantially to the treatment landscape, although real-world utilization patterns may vary by region. For example, between November 2022 and July 2023 in the United States, rituximab accounted for 53% of NMOSD treatments, followed by satralizumab (8%), eculizumab (7%), and inebilizumab (4%) (21).

Complement pathway inhibition was the second most common approach (17.6%), equally represented by eculizumab and ravulizumab (8.8% each). NMOSD is driven by AQP4-IgG-mediated astrocytopathy, which triggers the classical complement cascade, leading to astrocyte destruction, demyelination, and disability. This pathogenic mechanism underlies the robust efficacy of C5 blockade in preventing relapses (22). Pivotal placebo-controlled studies have confirmed the benefit of C5 inhibition: the PREVENT trial established eculizumab’s efficacy in AQP4-IgG-positive adults and secured the first FDA approval for NMOSD in 2019, while the 2024 FDA approval of long-acting ravulizumab (CHAMPION-NMOSD) expanded therapeutic options and reaffirmed the value of sustained complement inhibition.

Conventional immunosuppressants (16.5%) remain clinically relevant, with azathioprine, mycophenolate mofetil, and mitoxantrone each representing 4.4%, and interferon-β accounting for 3.3%. Their effectiveness, however, is limited; in one retrospective cohort, only 55.9% of AZA-treated and 50% of MMF-treated patients remained relapse-free during follow-up (23). Moreover, the variable or even adverse outcomes observed with interferon-β underscore the immunological distinctions between NMOSD and multiple sclerosis, highlighting the risks of non-targeted cytokine modulation (24).

Glucocorticoids (11.0%), particularly methylprednisolone (8.8%), were commonly used for acute management, providing rapid control of inflammation but lacking disease-modifying properties for long-term relapse prevention.

Targeting the IL-6 pathway accounted for 10.9% of interventions, represented by satralizumab. IL-6 plays a central role in NMOSD pathogenesis, with elevated levels in both serum and cerebrospinal fluid. It promotes plasma cell survival, enhances AQP4-IgG production, disrupts blood–brain barrier integrity, and supports proinflammatory T-cell activation. By blocking the IL-6 receptor, satralizumab interferes with these key pathogenic processes, attenuating the inflammatory environment that sustains AQP4 autoimmunity (25, 26).

Other strategies (6.6%), including autologous hematopoietic stem cell transplantation (HSCT) and immunoglobulin therapy, added further diversity to the therapeutic arsenal. HSCT has been employed in refractory cases, with a PRISMA-compliant meta-analysis demonstrating a favorable safety profile for intermediate-intensity regimens (27). While its rapid onset of action is advantageous, the risks of severe infections and prolonged immunosuppression, coupled with limited long-term efficacy data, constrain its broader application.

Finally, Bruton tyrosine kinase (BTK) inhibition (3.3%)-exemplified by edralbrutinib-represents an emerging upstream strategy. By modulating B-cell receptor signaling and Fc-mediated myeloid activation, BTK inhibitors aim to disrupt early drivers of disease, reflecting the field’s progression toward more targeted interventions (24).

Collectively, the therapeutic landscape mirrors the evolving understanding of NMOSD pathobiology. Current and investigational approaches converge on three primary objectives: (i) eliminating or suppressing AQP4-IgG-producing cells via CD19/CD20 depletion or BTK inhibition, (ii) neutralizing the downstream complement-mediated effector mechanisms through C5 blockade, and (iii) mitigating the IL-6-driven environment that sustains plasmablast survival and blood-brain barrier disruption. This tiered strategy from upstream prevention of autoantibody generation to downstream protection against astrocytic injury provides a coherent framework for both present and future NMOSD therapies (22).

The predominance of safety-related endpoints adverse events in 40 trials, with safety/tolerability and serious adverse events following closely reflects ongoing regulatory caution and echoes past safety concerns, notably meningococcal infections linked to eculizumab (28). Although relapse metrics such as relapse rate (22 trials) and time to relapse (10 trials) remain the cornerstone of efficacy assessment, this emphasis comes at the expense of outcomes that matter directly to patients. Disability progression by EDSS (19 trials) is still the most common functional endpoint, despite its insensitivity to visual pathway involvement. Only four trials evaluated visual acuity, and none included validated vision-specific patient-reported outcomes (e.g., NEI-VFQ-25), despite FDA recommendations in this area (29).

Pharmacokinetic measures such as AUC (17 trials) and Cmax (14 trials) were frequently included, aligning with monitoring needs for biologics (30). Yet neuroimaging endpoints were rare: just four trials incorporated MRI, overlooking evidence that silent MRI activity may predict future relapse (31). Likewise, no studies examined fluid biomarkers such as GFAP or neurofilament light chain, which are increasingly recognized as markers of astrocyte and axonal injury. Cardiac telemetry, included in six trials, likely reflects vigilance around specific risks such as satralizumab-associated arrhythmias, but beyond that, biomarker adoption remains minimal.

Overall, the current endpoint landscape still leans heavily toward safety monitoring and relapse prevention. While understandable from a regulatory standpoint, this narrow focus risks underrepresenting the broader disease burden. Expanding outcome measures to include visual function, quality-of-life instruments, and biomarker-based readouts would provide a more complete picture of NMOSD and align trial design more closely with patient needs and evolving pathobiological insights (32). Relapse-related endpoints should remain central for late-phase efficacy, but future trials should more routinely include vision-specific outcomes and validated patient-reported measures. Biomarkers (e.g., silent MRI lesions, GFAP, NfL, and AQP4-IgG-related measures) are currently best positioned as secondary/exploratory endpoints to support mechanism and risk stratification; in early-phase studies, they may also serve as pharmacodynamic readouts to prioritize candidates for larger trials.

## 5 Conclusions

This review of 141 registered NMOSD pharmacotherapy trials provides an integrated picture of a rapidly expanding research field. Trial initiations increased sharply after 2018, coinciding with regulatory successes of targeted biologics. Although Phase I studies predominate, a meaningful proportion of Phase III programs and post-marketing investigations reflects both mechanistic diversification and a growing emphasis on long-term safety monitoring. However, interpretation of the landscape requires careful attention to trial design heterogeneity and AQP4-IgG enrollment populations, as phase labels alone do not fully capture evidentiary strength. Limited public results reporting remains a major barrier to evidence synthesis, and rare disease recruitment constraints continue to impede trial completion. Therapeutic programs remain centered on B-cell depletion and complement inhibition, with increasing incorporation of IL-6 receptor blockade and emerging strategies such as BTK inhibition. Primary endpoints remain heavily skewed toward safety and relapse measures; broader adoption of visual function outcomes, patient-reported measures, and biomarkers would better align trials with patient priorities and disease biology.

## Supplementary material

Supplementary Figure 1 Flowchart of trial identification and selection.
