# Supplementary figures and images for: Global landscape of neuromyelitis optica spectrum disorder clinical trials: trends in therapies, geography, and outcomes
Source: Front Immunol. 2026 Apr 15;17:1695727. doi: 10.3389/fimmu.2026.1695727 (PMC13124598; doi:10.3389/fimmu.2026.1695727)

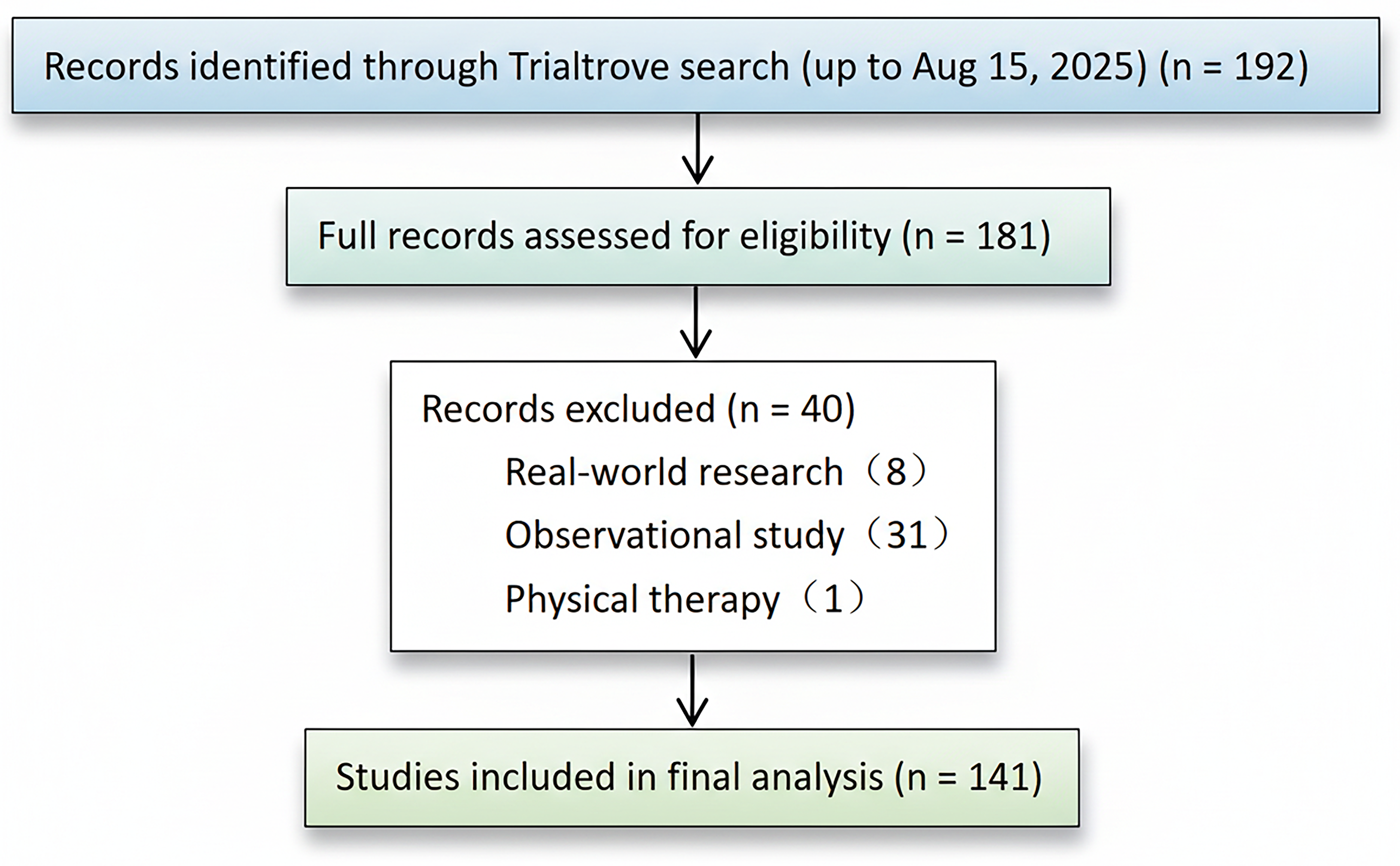

Supplement: Supplementary Figure 1 — Flowchart of trial identification and selection. [file Image1.png]
